# Two step I to II type transitions in layered Weyl semi-metals and their impact on superconductivity

**DOI:** 10.1038/s41598-023-35704-5

**Published:** 2023-05-25

**Authors:** Baruch Rosenstein, B. Ya. Shapiro

**Affiliations:** 1 Department of Electrophysics, National Yang Ming Chiao Tung University, Hsinchu, Taiwan, R.O.C.; 2 Department of Physics, Institute of Superconductivity, Bar-Ilan University, 52900 Ramat Gan, Israel

**Keywords:** Superconducting properties and materials, Physics

## Abstract

Novel “quasi two dimensional” typically layered (semi) metals offer a unique opportunity to control the density and even the topology of the electronic matter. Along with doping and gate voltage, a robust tuning is achieved by application of the hydrostatic pressure. In Weyl semi-metals the tilt of the dispersion relation cones, $\kappa ,$ increases with pressure, so that one is able to reach type II ($\kappa >1$starting from the more conventional type I Weyl semi-metals $\kappa <1$. The microscopic theory of such a transition is constructed. It is found that upon increasing pressure the I to II transition occurs in two continuous steps. In the first step the cones of opposite chirality coalesce so that the chiral symmetry is restored, while the second transition to the Fermi surface extending throughout the Brillouin zone occurs at higher pressures. Flattening of the band leads to profound changes in Coulomb screening. Superconductivity observed recently in wide range of pressure and chemical composition in Weyl semi-metals of both types. The phonon theory of pairing including the Coulomb repulsion for a layered material is constructed and applied to recent extensive experiments on $HfTe_{5}$.

## Introduction

The 3D and 2D topological quantum materials, such as topological insulators and Weyl semi-metals (WSM), attracted much interests due to their rich physics and promising prospects for applications. The band structure in the so called type I WSM like graphene^1^ in 2D and $ZrTe_{5}$ in 3D^2–14^, is characterized by appearance of linear dispersion relation, cones around several Dirac points, due to the band inversion. This is qualitatively distinct from conventional metals, semi-metals or semiconductors, in which bands are typically parabolic. The dispersion cones are often tilted^15–17^. In an extreme case of type-II WSMs, the cones have such a strong tilt, $\kappa \ge 1$, that they exhibit a nearly flat band at Fermi surface first predicted^18^ in $WTe_{2}$. Typically the Fermi surface “encircles” the Brillouin zone and therefore is topologically distinct from conventional “pockets”. This in turn leads to exotic electronic properties different from conventional and the type I materials. Examples include the collapse of the Landau level spectrum in magnetoresistance^19^, and novel quantum oscillations^20^. Several *layered *materials were predicted and observed to undergo^21^ the I to II (abbreviated as $I\rightarrow II$) transition while doping or pressure is changed^22–25^. In fact a well known layered organic compound $\alpha -(BEDT-TTF)_{2}I_{3}$ was a long time suspected^15–17^ to be a quasi—2D materials undergoing such transition.

Recent experiments concentrated on two (close) families of layered materials. The first is superlattice of transition metal dichalcogenides^26–31^layers with formula $MX_{2}$. The metals include $M=Mo,W,V,Ta,Pd$ , and the chalcogenides $X=S,Te,Se$. Majority of representatives of these class are 2D WSM. The well separated layers are integrated into van der Waals heterostructures by vertically stacking^32,33^. Intercalation and external pressure are the direct and effective methods for achieving exotic properties distinctive from the pristine materials^34–36^. Yet another class of stacked transition metal pentatellurides, including $HfTe_{5}$ and $ZrTe_{5}$, were recently comprehensively investigated^10,37–40^. For example the transport and superconductive properties of $HfTe_{5}$ were comprehensively studied^40^ at pressures as high as 30 GPa.

Pressure in particular^41^ controls both the strength of the interlayer coupling and of the cone slope allows to observe the topological transition. The affect on physical properties of the topological phase transitions between the type I to type II Weyl phases was considered theoretically. In ref.^42^ the heat capacity, compressibility and magnetic susceptibility was studied. Superconductivity observed recently in wide range of pressure and chemical composition in Weyl semi-metals of both types. In the previous paper^43^ and a related work^44^ a continuum theory of conventional superconductivity through the $I\rightarrow II$ topological transition was developed. Magnetic response in the superconducting state were calculated in^45,46^. The continuum approach used was too “mesoscopic” in order to describe the transition region since the global topology of the Brillouin zone is beyond the scope of the continuum approach.

In the present paper a theory of the topological transitions of the electron liquid of layered WSM under hydrostatic pressure is constructed using a (microscopic) tight binding model on the honeycomb lattice similar to that used to model^47^ dichalcogetite 2*H* $WTe_{2}$. It possesses an important chiral symmetry between two Brave (hexagonal) sublattices. The Weyl cones of opposite chirality appear at the crystallographic *K* and $K^{\prime }$ points for $\kappa =0$. The (discrete) chiral symmetry persists at all values of $\kappa$. This relatively simple model describes well both classes of layered materials that are Weyl semimetals.

Unexpectedly investigation of the pressure—“topology” phase diagram of this sufficiently universal microscopic model reveals that (at nonzero chemical potential) the $I\rightarrow II$ transition always occurs in two steps. In the first step upon increasing pressure leading to higher tilt $\kappa$ the circular pockets around the cones of opposite chirality coalesce into a single (type I) elliptic Fermi surface. The chiral symmetry is spontaneously broken. The second transition to the type II Fermi surface (extending throughout the Brillouin zone) occurs at yet higher pressures.

As in previous investigations^43,44^ superconductivity is used as an efficient signature of the topological transition. The phonon pairing theory was improved compared to previous work by accounting for the effects of screened Coulomb repulsion. We calculate the superconducting critical temperature taking into consideration the modification of the Coulomb electron-electron interaction. The Gorkov equations for two sublattics system are solved without resorting to the mesoscopic approach. Moreover it turns out that the screening of Coulomb repulsion plays a much more profound role in quasi 2D materials and do not allow the pseudo-potential simplification developed by MacMillan^48^. Taking this into account involves a nontrivial dependence on quasi-momentum in the gap equation (along with frequency dependence). The results compare well with recent experiment on^40^ $HfTe_{5}$.

Rest of the paper is organized as follows. In “Methods or procedure” Section the universal microscopic model of the layered WSM is described. The dependence of the tilt parameter $\kappa$, electron density and the interlayer distance on pressure are phenomenologically related to parameters of the model. In “Two step I to II type topological transition” Section the Gorkov equations for the optical phonon mediated intra-layer pairing for a multiband system including the Coulomb repulsion is derived and solved numerically. In Section “Inter-layer hopping on honeycomb lattice” the phonon theory of pairing including the Coulomb repulsion for a layered material is applied to recent extensive experiments on $HfTe_{5}$ under the hydrostatic pressure. The last Section contains “Conclusions” and discussion.

## Method or procedure

### A “universal” lattice model of layered (type I and type II) Weyl semi-metals

#### Inter-layer hopping on honeycomb lattice

A great variety of tight binding models were used to describe Weyl (Dirac) semimetals in 2D. Historically the first was graphene (type I, $\kappa =0$) , in which electrons hope between the neighboring cites of the honeycomb lattice. Two Dirac cones appear at *K* and $K^{\prime }$ crystallographic points in Brillouin one (BZ). Upon modification (gate voltage, pressure,intercalation) the hexagonal symmetry is lost, however a discrete chiral symmetry between two sublattices, denoted by $I=A,B$, ensures the 2D WSM. The tilted type I and even type II ($\kappa >1$) WSM can be described by the same Hamiltonian with the tilt term added. We restrict the discussion to systems with the minimal two cones of opposite chirality and negligible spin orbit coupling. This model describes the compounds listed in Introduction and can be generalizable to more complicated WSM. This 2D model is extended to a layered system with interlayer distance *d*. The 2D WSM layers are separated by dielectric streaks with interlayer hopping neglected, so that they are coupled electromagnetically only^49,50^.

The lateral atomic coordinates on the honeycomb lattice are ${\textbf{r}}_{ {\textbf{n}}}=n_{1}{\textbf{a}}_{1}+n_{2}{\textbf{a}}_{2}$, where lattice vectors are:

1 $$\begin{aligned} {\textbf{a}}_{1}=a\left( \frac{1}{2},\frac{\sqrt{3}}{2}\right) ;{\textbf{a}}_{2}=a\left( \frac{1}{2},-\frac{\sqrt{3}}{2}\right) \text {.} \end{aligned}$$

    The length of the lattice vectors *a* will be taken as the length unit and we also set $\hbar =1$. The hopping Hamiltonian including the tilt term is:

2 $$\begin{aligned} K=\sum \nolimits _{{\textbf{n}}l}\left\{ t\left( \sum \limits _{i=1,2,3}\psi _{ {\textbf{n}}l}^{sA\dagger }\psi _{{\textbf{r}}_{{\textbf{n}}}+\mathbf {\delta } _{i},l}^{sB}+\mathrm {h.c.}\right) -\kappa \psi _{{\textbf{n}}l}^{sI\dagger }\psi _{{\textbf{r}}_{{\textbf{n}}}+{\textbf{a}}_{1},l}^{sI}-\mu n_{{\textbf{n}},l}\right\} \text {.} \end{aligned}$$

    Here an integer *l* labels the layers. Operator $\psi _{{\textbf{n}} l}^{sA\dagger }$ is the creation operators with spin $s=\uparrow ,\downarrow$, while the density operator is defined as $n_{{\textbf{n}}l}=\psi _{\textbf{n }l}^{sI\dagger }\psi _{{\textbf{n}}l}^{sI}$. The chemical potential is $\mu$, while *t* is the hopping energy. Each site has three neighbors separated by vectors $\mathbf {\delta }_{1}=\frac{1}{3}\left( {\textbf{a}}_{1}-{\textbf{a}} _{2}\right) ,\mathbf {\delta }_{2}=-\frac{1}{3}\left( 2{\textbf{a}}_{1}+\textbf{ a}_{2}\right)$ and $\mathbf {\delta }_{3}=\frac{1}{3}\left( {\textbf{a}}_{1}+2 {\textbf{a}}_{2}\right)$. Dimensionless parameter $\kappa$ determines the tilt of the Dirac cones along the ${\textbf{a}}_{1}$direction^15–17^. In the 2D Fourier space, $\psi _{n_{1}n_{2}l}^{sA\dagger }=N_{s}^{-2}\sum \nolimits _{k_{1}k_{2}}\psi _{k_{1}k_{2}l}^{sA\dagger }\exp \left[ 2\pi i\left( k_{1}n_{1}\mathbf {+}k_{2}n_{2}\right) /N_{s}\right]$, one obtains for Hamiltonian

(for finite discrete reciprocal lattice $N_{s}\times N_{s}$):

3 $$\begin{aligned} K=\frac{1}{N_{s}^{2}}\sum \nolimits _{k_{1}k_{2}l}\psi _{k_{1}k_{2}l}^{s\dagger }M_{k_{1}k_{2}}\psi _{k_{1}k_{2}l}^{s}\text {.} \end{aligned}$$

    Here ${\textbf{k}}=\frac{k_{1}}{N_{s}}{\textbf{b}}_{1}+\frac{k_{2}}{N_{s}} {\textbf{b}}_{2}$ are the reciprocal lattice vectors and the matrix

4 $$\begin{aligned} M_{{\textbf{k}}}=d_{{\textbf{k}}}^{x}\sigma _{x}+d_{{\textbf{k}}}^{y}\sigma _{y}+d_{{\textbf{k}}}^{0}I \end{aligned}$$

where

5 $$\begin{aligned} d_{{\textbf{k}}}^{x}= & {} \cos \left[ \frac{2\pi }{3N_{s}}\left( k_{1}-k_{2}\right) \right] +2\cos \left[ \frac{\pi }{N_{s}}\left( k_{1}+k_{2}\right) \right] \cos \left[ -\frac{\pi }{3N_{s}}\left( k_{1}-k_{2}\right) \right] ; \nonumber \\ d_{{\textbf{k}}}^{y}= & {} -\sin \left[ \frac{2\pi }{3N_{s}}\left( k_{1}-k_{2}\right) \right] +2\cos \left[ \frac{\pi }{N_{s}}\left( k_{1}+k_{2}\right) \right] \sin \left[ \frac{\pi }{3N_{s}}\left( k_{1}-k_{2}\right) \right] ; \nonumber \\ d_{{\textbf{k}}}^{0}= & {} -\kappa \cos \left[ \frac{2\pi }{N_{s}}k_{1}\right] -\mu \text {.} \end{aligned}$$

    From now on the hopping energy *t* will be our energy unit.

The free electrons part of the Matsubara action for Grassmanian fields $\psi _{{\textbf{k}}ln}^{*sI}$ therefore is:

6 $$\begin{aligned} S^{e}=\frac{1}{T}\sum \nolimits _{{\textbf{k}}ln}\psi _{{\textbf{k}}ln}^{*sA}\left\{ \left( -i\omega _{n}+d_{{\textbf{k}}}^{0}\right) \delta ^{AB}+\sigma _{i}^{AB}d_{{\textbf{k}}}^{i}\right\} \psi _{{\textbf{k}}ln}^{sB} \text {.} \end{aligned}$$

Here $\omega _{n}=\pi T\left( 2n+1\right)$ is the Matsubara frequency. The Greens’ function, $g_{{\textbf{k}}n}^{ss^{\prime }}=\delta ^{ss^{\prime }}g_{ {\textbf{k}}n}$, of free electrons has the (sublattice) following matrix form:

7 $$\begin{aligned} g_{{\textbf{k}}n}=\left[ \left( -i\omega _{n}+d_{{\textbf{k}}}^{0}\right) I+\sigma _{i}d_{{\textbf{k}}}^{i}\right] ^{-1}=\frac{\left( -i\omega _{n}+d_{ {\textbf{k}}}^{0}\right) I-\sigma _{i}d_{{\textbf{k}}}^{i}}{\left( i\omega _{n}-d_{{\textbf{k}}}^{0}\right) ^{2}-\left( d_{{\textbf{k}}}^{x2}+d_{{\textbf{k}} }^{y2}\right) }\text {.} \end{aligned}$$

    Now we turn to the interactions part of the Hamiltonian.

### Coulomb repulsion

The electron-electron repulsion in the layered WSM on the lattice can be presented in the form,

8 $$\begin{aligned} V=\frac{e^{2}}{2}\sum \nolimits _{\textbf{nn}^{\prime }ll^{\prime }}n_{\textbf{ n}l}v_{\mathbf {n-n}^{\prime },l-l^{\prime }}^{C}n_{{\textbf{n}}^{\prime }l^{\prime }}\text {,} \end{aligned}$$

where $v_{\mathbf {n-n}^{\prime },l-l^{\prime }}^{C}$ is the “bare” Coulomb interaction between electrons. Making the 2D Fourier transform, one obtains,

9 $$\begin{aligned} V=\frac{e^{2}}{2N_{s}^{2}}\sum \nolimits _{{\textbf{q}}ll^{\prime }}n_{{\textbf{q}} l}v_{\textbf{q,}l-l^{\prime }}^{C}n_{-{\textbf{q}}l^{\prime }}\text {,} \end{aligned}$$

where

10 $$\begin{aligned} v_{{\textbf{q}},l-l^{\prime }}^{C}=v_{{\textbf{q}}}^{2D}e^{-dq\left| l-l^{\prime }\right| }\text {,} \end{aligned}$$

with the in plane Coulomb repulsion being $v_{{\textbf{q}}}^{2D}=\frac{2\pi e^{2}}{q\epsilon }$. Here $\epsilon$ is the inter-layer dielectric constant^59^, while *d* is the interlayer distance. On the hexagonal lattice the exponential formula approximates the Coulomb repulsion well only away from the BZ boundaries . The long range screening effect of the Coulomb interaction is effectively taken into account using the RPA approximation. Effect of pressure on the various parameters is discussed in the next section.

## Two step I to II type topological transition

### Pressure induced parameter modifications

While pressure turned out to be more experimentally accessible control parameter than the gate voltage, in the early works mentioned in Introduction typically the phase diagram was studied as a function of the chemical potential. Moreover in most recent experiments the hydrostatic pressure serves as a control parameter to induce topological transformations of the electronic matter in WSM. The parameter dependence of a microscopic model on pressure, is in principle derivable by the DFT and a corresponding adaptation of the elasticity theory^41^. Although there exist a qualitative theoretical description of the pressure dependence of the Coulomb repulsion^51^, electron-phonon coupling and the topology of the Fermi surface of these novel materials^52,53^, it is difficult to determine quantitatively the tilt $\kappa$, inter layer spacing *d*, electron density and other parameters. Therefore we use an experimentally parametrized (see for example a comprehensive study^54^) dependence of these parameters on the pressure. In the present paper to describe a specific material $HfTe_{5}$ as an example we utilize experimental results of ref.^40^. Note that in many materials the robust electron gas exists only at certain pressure.

For not very large pressures ($P<15$ GPa) several parameters dependencies can be accounted for as linear. In particular, the layer spacing and the tilt parameter are modified under pressure *P* as:

11 $$\begin{aligned} d\left( P\right)=\,& {} \frac{d_{a}}{1+\sigma P/d_{a}}\approx d_{a}-\sigma P\text {;} \nonumber \\ \kappa \left( P\right)=\,& {} \kappa _{a}+\gamma P\text {.} \end{aligned}$$

    The tilt parameter was estimated in ref.^41^ for a wide range of $\kappa$. For layered $HfTe_{5}$ the stress parameter is $\sigma = 0.225$ A/GPa. The “ambient” value is $d_{a}=7.7A$. As noted above the electron gas exists^40^ in this case only for $P>3$ GPa. For the tilt modulus $\kappa _{a}=-0.3$ and $\gamma =0.15/$GPa.

Measurements demonstrate that 3D electron density in the type I phase of layered WSM is exponential in pressure (for not very high pressures):

$$\begin{aligned} n^{3D}\left( P\right) =n_{a}e^{\beta P}\text {.} \end{aligned}$$

    It saturates upon approach to type II WSM. The ambient value is $n_{a}=1.4\times 10^{19}$ cm$^{-3}$, while $\beta =0.77/$GPa. The two dimensional electron density in the layers is related to the measured density by $n\left( P\right) =n^{3D}\left( P\right) d\left( P\right)$. The influence on the interactions will be discussed in the next Section. Having described the model let us turn to the spectrum and topology of the Fermi surface for different pressures.

### Topological phases of layered WSM

**Figure 1:**
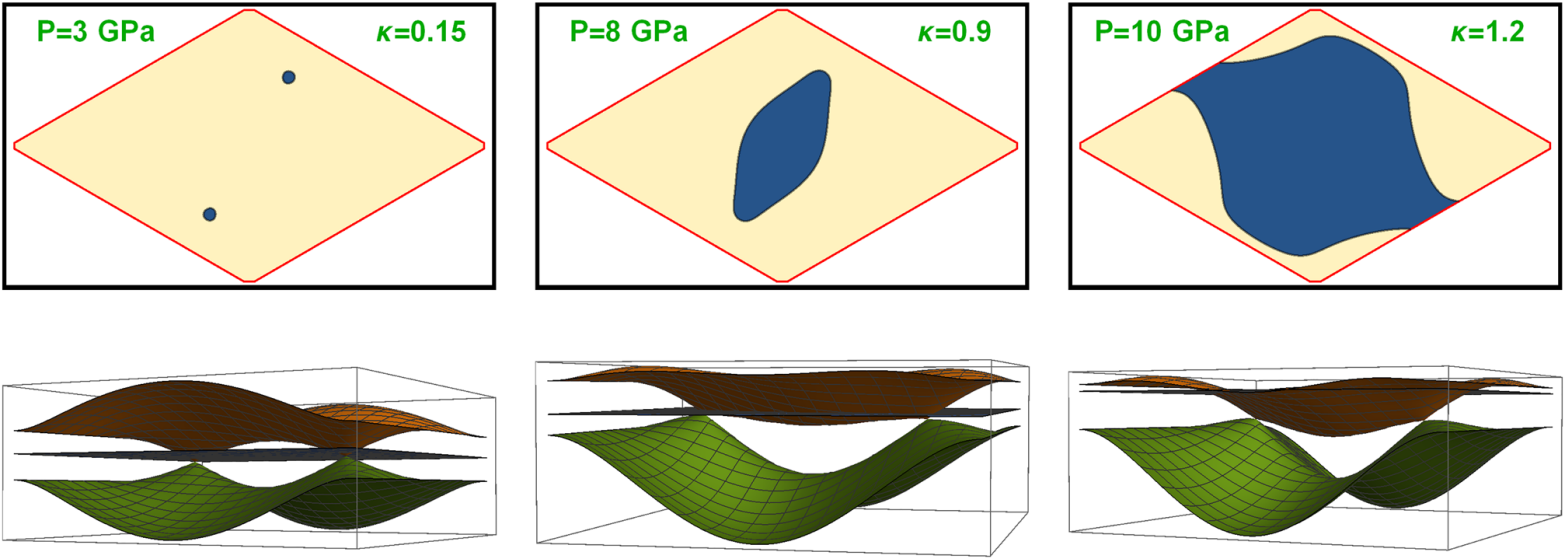
Evolution of the Fermi surface topology as the pressure of Weyl semimetal increase. Parameters like the tilt $\kappa \left( P\right)$, electron density etc are given in Eq. (11) of the Weyl semimetal. The upper raw depicts the Fermi surfaces of all three topological phases, while the lower row are the corresponding dispersion relation of both branches (brown and green surfaces) with respect to Fermi level (the blue plane). At relatively low pressure the FS consists of two small Dirac pockets. At intermediate pressures the two pockets merge into a single ellipsoidal large pocket (still type I). At very high pressures the electron liquid undergoes the type I to type II topological transition.

Upon increasing pressure the I to II transition occurs in two continuous steps. In the first step the cones of opposite chirality coalesce so that the chiral symmetry is restored, while the second transition to the Fermi surface extending throughout the Brillouin zone occurs at higher pressures. Figure 1 describes the Fermi surface (blue areas depict the Fermi sea in upper contour plots) and dispersion relations (lower 3D plots) of three representative pressures value from the three phases. There are two branches (brown higher than green) crossing the Fermi level (blue plane).

The graphene-like dispersion relation for smallest value of pressure when the electron pockets exist, $P=3$ GPa, $\kappa =0.15$ (left panel in Fig. 1) represents the type I WSM below the chiral transition. A rhombic BZ (with coordinates $k_{1}$ and $k_{2}$ defined in Eq. (3), yellow area covers the BZ) is chosen. Location of the cones (see a lower 3D plot) are close to crystallographic $K^{\pm }$ points. There are two slightly tilted Dirac cones of opposite chirality. Increasing the pressure towards the chiral transition at $P_{\chi }=5.8$ GPa, the two pockets of the Fermi surface become elongated and larger and eventually merge into a single pocket shown in the central figure for $P=8$ GPa. The tilt parameter is already significant $\kappa =0.9$. At yet larger pressure $P=10$ GPa (right panel) the material becomes a type II WSM with large $\kappa >1.2$. In this case FS envelops the BZ that topologically is torus. See the segment on the boundary $k_{2}=0=2\pi /a$. Obviously the upper band becomes flatter as the tilt (pressure) increases.

**Figure 2:**
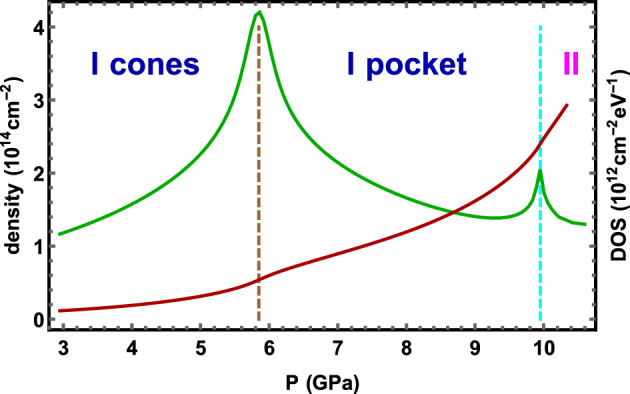
Electron density and density of states (DOS) as function of pressure *P*.of WSM. The 2D electron density (the brown curve) monotonically increases, while DOS (the green curve) has cusps at both topological transitions. On the cusp the derivative of DOS with respect pressure changes sign.

Figure 2 gives the 2D electron density and the density of states as function of the pressure for Hamiltonian of the previous Section.

Both the electron density and the density of states were calculated numerically for the Fermi distribution function at temperature $T=1$K (the density at zero temperature corresponds to an area inside the FS) at various values of the chemical potential. Then the density is matched with those determined phenomenologically in the previous subsection.

### The first topological transition: spontaneous chiral symmetry breaking

At small pressures, 3 GPa $<P<4$ GPa, the Fermi surface consists of two well separated Dirac cones of opposite chirality. The tilt does not affect the basic chiral symmetry of the honeycomb lattice: two sublattices are related by a reflection. The sixfold symmetry in undistorted graphene is of course typically broken down to the reflection symmetry only. When the tilted cones FS pockets merge at the transition $P=P_{\chi }=5.8$ GPa (see the brown line in Fig. 2) the chiral symmetry of the ground state is restored. The overall chirality of the FS above $P_{\chi }$ (a topological number) therefore is zero. Although we are not aware of a mathematical proof, this transition always precedes the $I\rightarrow II$ topological transition, see cyan line in Fig. 2. The chiral transition is also topological, but a more local sense: fracture of the Fermi surface like in graphene oxide^50^ or Lifshitz transition in high $T_{c}$ cuprates like $La_{2-x}Sr_{x}CuO_{4}$. The $I\rightarrow II$ is more “exotic”^55^: it involves the global topology of the Fermi surface (it is a torus). The DOS at transition (the green curve in Fig. 2) has a finite maximum at which the derivative changes sign. The reason of the DOS increase is caused by change of the dispersion relation of two merging Weyl fermions with opposite chirality. In this case the fermions dispersion relation which is linear along two directions becomes linear along one direction and quadratic along another one. In this case the DOS increases dramatically^56–58^.

### The second topological transition: ${\rm I \rightarrow II}$

The electron density in type I phase above the chiral transition grows quite fast, see red line in Fig. 2, so that at large pressures a significant part of BZ for one of the branches of spectrum is occupied. Eventually at $P_{I\rightarrow II}=9.9$ GPa the growing single pocket envelops the BZ torus and thus FS splits again into two curves, see the right panel in Fig. 1. Density of electron saturates, while the DOS has another finite peak. The two transition lead to singularities in various physical quantities. In the next Section the screening of Coulomb interactions is discussed.

## Screening in layered Weyl semi-metal

The screening in the layered system can be conveniently partitioned into the screening within each layer described by the polarization function $\Pi _{ {\textbf{q}}n}$ and electrostatic coupling to carriers in other layers. We start with the former.

### Polarization function of the electron gas in Layered WSM

In a simple Fermi theory of the electron gas in normal state with Coulomb interaction between the electrons in RPA approximation the Matsubara polarization is calculated as a simple *minus* “fish” diagram^49^ in the form:

12 $$\begin{aligned} \Pi _{{\textbf{q}}n}=2T\sum \nolimits _{{\textbf{p}}m}\text {Tr}\left[ g_{{\textbf{p}} m}g_{\mathbf {p+q},m+n}^{tr}\right] \text {.} \end{aligned}$$

    Using the GF (see Eq. 7), one obtain:

13 $$\begin{aligned} \Pi _{{\textbf{q}}n}=\frac{4T}{N_{s}^{2}}\sum \nolimits _{{\textbf{p}}m}\frac{ \left( i\omega _{m}+A\right) \left( i\omega _{m}+B\right) +C}{\left[ \left( i\omega _{m}+A\right) ^{2}-\alpha ^{2}\right] \left[ \left( i\omega _{m}+B\right) ^{2}-\beta ^{2}\right] }\text {,} \end{aligned}$$

where

14 $$\begin{aligned} A= & {} -d_{{\textbf{p}}}^{0};B=i\omega _{n}-d_{\mathbf {p+q}}^{0};C=d_{{\textbf{p}} }^{x}d_{\mathbf {p+q}}^{x}-d_{{\textbf{p}}}^{y}d_{\mathbf {p+q}}^{y} \nonumber \\ \alpha ^{2}= & {} d_{{\textbf{p}}}^{x2}+d_{{\textbf{p}}}^{y2};\beta ^{2}=d_{\mathbf { p+q}}^{x2}+d_{\mathbf {p+q}}^{y2}\text {.} \end{aligned}$$

    Performing summation over *m*, one obtains:

15 $$\begin{aligned} \Pi _{{\textbf{q}}n}=-\frac{1}{N_{s}^{2}}\sum \nolimits _{{\textbf{p}}}\left\{ \begin{array}{c} \frac{\alpha ^{2}-\alpha (A-B)+C}{\alpha \left[ \left( A-B-\alpha \right) ^{2}-\beta ^{2}\right] }\tanh \frac{\alpha -A}{2T}+\frac{a^{2}+\alpha (A-B)+C }{\alpha \left[ \left( A-B+\alpha \right) ^{2}-\beta ^{2}\right] }\tanh \frac{\alpha +A}{2T} \\ +\frac{\beta ^{2}+\beta \left( A-B\right) +C}{\beta \left[ \left( A-B+\beta \right) ^{2}-\alpha ^{2}\right] }\tanh \frac{\beta -B}{2T}+\frac{\beta ^{2}-\beta \left( A-B\right) +C}{\beta \left[ \left( A-B-\beta \right) ^{2}-\alpha ^{2}\right] }\tanh \frac{\beta +B}{2T} \end{array} \right\} \text {.} \end{aligned}$$

    The polarization function however is strongly differ from the usual Lindhard expression for a parabolic band.

### Screening due to electron gas in layered system

Coulomb repulsion between electrons in different layers *l* and $l^{\prime }$ within the RPA approximation is determined by the following integral equation:

16 $$\begin{aligned} V_{\textbf{q,}l-l^{\prime }\textbf{,}n}^{RPA}=v_{{\textbf{q}},l-l^{\prime }}^{C}+\Pi _{{\textbf{q}}n}\sum \nolimits _{l^{\prime \prime }}v_{{\textbf{q}},l-l^{\prime \prime }}^{C}V_{{\textbf{q}},l^{\prime \prime }-l^{\prime } \textbf{,}n}^{RPA}. \end{aligned}$$

    The polarization function $\Pi _{{\textbf{q}}n}$ in 2D was calculated in the previous subsection. This set of equations is decoupled by the Fourier transform in the *z* direction:

17 $$\begin{aligned} V_{\textbf{q,}q_{z},n}^{RPA}=\frac{v_{{\textbf{q}},q_{z}}^{C}}{1-\Pi _{\textbf{ q}n}v_{{\textbf{q}},q_{z}}^{C}}\text {, } \end{aligned}$$

where

18 $$\begin{aligned} v_{{\textbf{q}},q_{z}}^{C}=\sum \nolimits _{l}v_{{\textbf{q}}}^{2D}e^{iq_{z}l-qd \left| l\right| }=v_{{\textbf{q}}}^{2D}\frac{\sinh \left( qd\right) }{ \cosh \left( qd\right) -\cos \left( dq_{z}\right) }\text {.} \end{aligned}$$

    The screened interaction in a single layer therefore is is given by the inverse Fourier transform^49^:

19 $$\begin{aligned} V_{\textbf{q,}l-l^{\prime },n}^{RPA}=\frac{d}{2\pi }\int _{q_{z}=-\pi /d}^{\pi /d}e^{iq_{z}d\left( l-l^{\prime }\right) }\frac{v_{{\textbf{q}} q_{z}}^{C}}{1-\Pi _{{\textbf{q}}n}v_{{\textbf{q}}q_{z}}^{C}}\text {.} \end{aligned}$$

    Considering screened Coulomb potential at the same layer $l = l^{\prime } ,$ the integration gives,

20 $$\begin{aligned} V_{{\textbf{q}}n}^{RPA}=\frac{v_{{\textbf{q}}}^{2D}\sinh \left[ qd\right] }{ \sqrt{b_{{\textbf{q}}n}^{2}-1}}, \end{aligned}$$

where $b_{{\textbf{q}}n}=\cosh \left( dq\right) -v_{{\textbf{q}}}^{2D}\Pi _{ {\textbf{q}}n}\sinh \left( dq\right)$. This formula is reliable only away from plasmons $b_{{\textbf{q}}n}>1$. It turns out that to properly describe superconductivity, one can simplify the calculation at low temperature by considering the static limit $\Pi _{{\textbf{q}}n}\simeq \Pi _{{\textbf{q}}0}$. Consequently the potential becomes static: $V_{{\textbf{q}}}^{RPA}\equiv V_{ {\textbf{q}},n=0}^{RPA}$.

## Superconductivity

Superconductivity in WSM is caused by a conventional phonon pairing. The leading mode is an optical phonon mode assumed to be dispersionless with energy $\Omega$. The effective electron-electron interaction due to the electron–phonon attraction opposed by Coulomb repulsion (pseudo-potential) creates pairing below $T_{c}$. Further we assume the singlet *s* -pairing channel and neglect the interlayer electrons pairing. It important to note that unlike in conventional 3D metal superconductors where a simplified pseudo-potential approach due to McMillan and other^48^, in 2D and layered WSM, one have to resort to a more microscopic approach.

### Effective attraction due to phonon exchange opposed by the effective Coulomb repulsion

The free and the interaction parts of the effective electron action (“integrating phonons”+RPA Coulomb interaction)^59,60^ in the quasi-momentum—Matzubara frequency representation, $S=S^{e}+S^{int}$,

21 $$\begin{aligned} S^{e}= & {} \frac{1}{T}\sum \limits _{{\textbf{k}},l,n}\psi _{{\textbf{k}}ln}^{*sA}\left\{ \left( -i\omega _{n}+d_{{\textbf{k}}}^{0}\right) \delta ^{AB}+\sigma _{i}^{AB}d_{{\textbf{k}}}^{i}\right\} \psi _{{\textbf{k}}ln}^{sB} \text {;} \nonumber \\ S^{int}= & {} \frac{1}{2T}\sum \nolimits _{{\textbf{q}}nn^{\prime }mm^{\prime }}n_{ {\textbf{q}}ln}\left( \delta _{ll^{\prime }}V_{\textbf{q,}m-m^{\prime }}^{ph}+V_{{\textbf{q}},l-l^{\prime }}^{RPA}\right) n_{-{\textbf{q}},-l^{\prime },-n^{\prime }}\text {.} \end{aligned}$$

    Here $n_{{\textbf{q}}ln}=\sum \nolimits _{{\textbf{p}}}\psi _{{\textbf{p}}ln}^{*sI}\psi _{\mathbf {q-p,}l,n}^{sI}$ the Fourier transform of the electron density. The effective electron - electron coupling due to phonons is:

22 $$\begin{aligned} V_{{\textbf{q}}m}^{ph}=-\frac{g^{2}\Omega }{\omega _{m}^{b2}+\Omega ^{2}}\text {,} \end{aligned}$$

where the bosonic frequencies are $\omega _{m}^{b}=2\pi mT$.

The pressure dependence on the frequency is approximated as:

23 $$\begin{aligned} \Omega \left( P\right) =\Omega _{a}\left( 1+\zeta P\right) . \end{aligned}$$

    For $HfTe_{5}$ we take $\Omega _{a}=15$ meV and $\zeta =0.005/$GPa.

### Nambu Green’s functions and Gorkov equations

Normal and anomalous (Matsubara) intra layer Nambu Green’s functions are defined by expectation value of the fields, $\left\langle \psi _{{\textbf{k}} nl}^{Is}\psi _{{\textbf{k}}nl}^{*s^{\prime }J}\right\rangle =\delta ^{ss^{\prime }}G_{{\textbf{k}}n}^{IJ}$ and $\left\langle \psi _{{\textbf{k}} nl}^{Is}\psi _{-\mathbf {k,-}n,l}^{Js^{\prime }}\right\rangle =\varepsilon ^{ss^{\prime }}F_{{\textbf{k}}n}^{IJ}$, while the gap function is

24 $$\begin{aligned} \Delta _{{\textbf{q}}n}^{IJ}=\sum \nolimits _{{\textbf{p}}m}V_{\mathbf {q-p,}n-m}F_{ {\textbf{p}}m}^{IJ}, \end{aligned}$$

where $V_{{\textbf{q}}n}=V_{{\textbf{q}}n}^{ph}+V_{{\textbf{q}}n}^{RPA}$ is a sublattice scalar. The gap equations in the sublattice matrix form are derived from Gorkov equations (see details in SI)^60^:

25 $$\begin{aligned} \Delta _{{\textbf{q}}n}=-\sum \nolimits _{{\textbf{p}}m}V_{\mathbf {q-p,}n-m}g_{ {\textbf{p}}m}\left\{ I+\Delta _{{\textbf{p}}m}g_{-{\textbf{p}},-m}^{t}\Delta _{- {\textbf{p}},-m}^{*}g_{{\textbf{p}}m}\right\} ^{-1}\Delta _{{\textbf{p}}m}g_{- {\textbf{p}},-m}^{t}\text {.} \end{aligned}$$

    This equation was solved numerically by iterations method. The momenta are discretized as $q_{1.2}$ $=2\pi j_{1,2}/N_{s}$ (where $j_{1,2}$ $=$ $-N_{s}/2$...$\left( N_{s}/2-1\right)$) $N_{s}=256$ while the frequency cutoff was $N_{T}=128$ the interatomic in-plane distance $a=3.5$ A, electron-phonon coupling $g=140$ meV and the dielectric constant $\varepsilon =20.$

**Figure 3:**
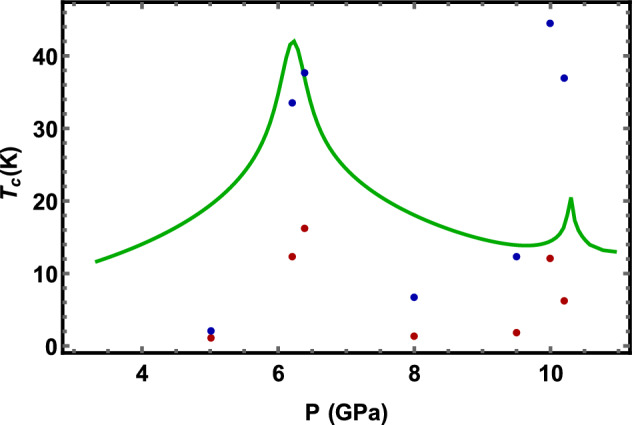
The critical temperature $T_{c}$ as function of the hydrostatic pressure *P* with (red points) and without (blue points) the Coulomb electron-electron interaction. The dependence has spikes near the points of topological transformations of the electronic system. Position of spikes coinsides with that of the density of states (the green curve).

The critical temperature as a function on the pressure is presented in Fig. 3. The blue points represent the $T_{c}$ when the Coulomb repulsion is neglected. It clearly shows the spikes of the $T_{c}$ near the points of the both topological transformation of the electronic system caused by the hydrostatic pressure. It amplifies the dependence of the density of states (green line) in these points that can be understood from the approximate exponential BCS dependence, $T_{c}=\Omega (P)e^{-D\left( P\right) g^{2}(P)}$ . A more realistic model includes the Coulomb repulsion, see red points in Fig. 3. The critical temperatures are much smaller demonstrating that in the present case the repulsion plays the essential role. It turns out that it not possible to approximate this behavior using a simplistic pseudo-potential approach by McMillan^48^ theory successfully applied to 3D good metals. It should be note that the shift of the $T_{c}$ from the maximum value of the DOS pressure dependence (see Fig. 2) is caused by the pressure increase of the electron phonons strength (see Eqs. 22, 23).

## Conclusion

To summarize we have developed a theory of superconductivity in layered Weyl semi-metals under the hydrostatic pressure that properly takes into account the Coulomb repulsion. It is shown that in Weyl semi-metals the tilt of the dispersion relation cones, $\kappa ,$ increases with pressure, so that one is able to reach type II ($\kappa >1$starting from the more conventional type I Weyl semi-metals , $\kappa <1$). It is found that upon increasing pressure the I to II transition occurs in two continuous steps. In the first step the cones of opposite chirality coalesce so that the chiral symmetry is restored, while the second transition to the Fermi surface extending throughout the Brillouin zone occurs at higher pressures. We show that the critical temperature is a very robust tool to study these transformations of the electronic system. The critical temperature shows spike in the points of topological transformation repeating the density of the electron states. The generalization goes beyond the simplistic pseudo-potential approach by McMillan^48^ theory. Superconductivity demonstrated significant effect of the Coulomb repulsion on the critical temperature.

## Supplementary Information

Supplementary Information.

## Data Availability

All data generated or analysed during this study are included in this published article [and its supplementary information files].
