# Supplementary material for: Two step I to II type transitions in layered Weyl semi-metals and their impact on superconductivity
Source: Sci Rep. 2023 May 25;13:8450. doi: 10.1038/s41598-023-35704-5 (PMC10213019; doi:10.1038/s41598-023-35704-5)
Supplement: Supplementary file 1 — Supplementary Information. [file 41598_2023_35704_MOESM1_ESM.pdf]

Part I
Supplemental Information

1 A. Details of the model

The system considered in the paper is fitted for the following values of the hipping and the tilt parameter. The hopping $t = 500\ meV$. Lattice symmetry length $a = 2.8A$ is unit of length. The calculations were performed on the discrete reciprocal lattice $k_1, k_2 = 1, ...N_s$ with $N_s = 256$. The in-plane structure is the honeycomb with atomic coordinates on the honeycomb lattice are $\mathbf{r_n} = n_1\mathbf{a}_1 + n_2\mathbf{a}_2$, where lattice vectors are:

$$\mathbf{a}_1 = a\left(\frac{1}{2}, \frac{\sqrt{3}}{2}\right);\ \mathbf{a}_2 = a\left(\frac{1}{2}, -\frac{\sqrt{3}}{2}\right). \tag{1}$$

The length of the lattice vectors a will be taken as the length unit and we set $\hbar = 1$ while the reciprocal lattice basis is

$$\mathbf{b}_1 = 2\pi\left(1, \frac{1}{\sqrt{3}}\right); \mathbf{b}_2 = 2\pi\left(1, -\frac{1}{\sqrt{3}}\right) \tag{2}$$

The Hamiltonian in Fourier is:

$$K = \frac{1}{N_s^2}\sum_{\mathbf{k},l}\psi^{s\dagger}_{\mathbf{k}l}M_{\mathbf{k}}\psi^{s}_{\mathbf{k}l} \tag{3}$$

In discrete reciprocal lattice $N_s \times N_s$

$$\mathbf{k} = \frac{k_1}{N_s}\mathbf{b}_1 + \frac{k_2}{N_s}\mathbf{b}_2; k_x = \frac{2\pi}{N_s}(k_1 + k_2), k_y = \frac{2\pi}{\sqrt{3}N_s}(k_1 - k_2); k_1, k_2 = 1, ...N_s \tag{4}$$

where

$$M_{\mathbf{k}} = \frac{\sqrt{3}}{2}\left\{\gamma\begin{pmatrix}0 & h\\ h^* & 0\end{pmatrix} + (-\gamma\kappa d_0 - \mu)\begin{pmatrix}1 & 0\\ 0 & 1\end{pmatrix}\right\} \tag{5}$$

where the structure function of links is

$$h = e^{i\delta_1\cdot\mathbf{k}} + e^{i\delta_2\cdot\mathbf{k}} + e^{i\delta_3\cdot\mathbf{k}} = ixp\left[k_y/\sqrt{3}\right] + ixp\left[-\frac{1}{2}k_x - \frac{k_y}{2\sqrt{3}}\right] + ixp\left[\frac{1}{2}k_x - \frac{k_y}{2\sqrt{3}}\right] \tag{6}$$

Using Pauli matrices this can be written as, $\gamma = 1$

$$M_{\mathbf{k}} = d_x\sigma_x + d_y\sigma_y + d_0 I \tag{7}$$

The one electron part of the Action has the form

$$S^e = \sum_{\mathbf{k},l,n} \psi^{*sA}_{\mathbf{k}l,n} \left\{ \left(-i\omega_n + d^0_{\mathbf{k}}\right) \delta^{AB} + \sigma_i^{AB} d^i_{\mathbf{k}} \right\} \psi^{sB}_{\mathbf{k},l,n}. \tag{8}$$

The inverse free Green Function can be represented in the matrix form as

$$g^{-1AB}_{0\mathbf{k}n} = \left(-i\omega_n + d^0_{\mathbf{k}}\right) \delta^{AB} + \sigma_i^{AB} d^i_{\mathbf{k}} \tag{9}$$

while the propagator

$$g_{\mathbf{k}n} = \frac{1}{\det_{\mathbf{k}n}} \begin{matrix} -i\omega_n + d^0_{\mathbf{k}} & -d^x_{\mathbf{k}} + id^y_{\mathbf{k}} \\ -d^x_{\mathbf{k}} - id^y_{\mathbf{k}} & -i\omega_n + d^0_{\mathbf{k}} \end{matrix} = \frac{1}{\det_{\mathbf{k}n}} \left(\left(-i\omega_n + d^0_{\mathbf{k}}\right) I - d^x_{\mathbf{k}} \sigma_x - d^y_{\mathbf{k}} \sigma_y \right) \tag{10}$$

Here $\det_{\mathbf{k}n} = \left(i\omega_n - d^0_{\mathbf{k}}\right)^2 - \left(d^{x2}_{\mathbf{k}} + d^{y2}_{\mathbf{k}}\right)$

The Coulomb electron-electron interaction is

$$V = \frac{1}{2} \sum_{\mathbf{n} \neq \mathbf{n}' l l'} n_{\mathbf{n}l} v^C_{\mathbf{n}-\mathbf{n}',l-l'} n_{\mathbf{n}'l'}, \tag{11}$$

where the Coulomb electron-electron interaction

$$v^C_{\mathbf{n}-\mathbf{n}',l} = \frac{e^2}{\sqrt{\mathbf{r}^2_{n-n'} + d^2 l^2}} \tag{12}$$

here d is the interlayer space, l is the number of the layer, $n_{\mathbf{n}l}$ is the electron density.

2 B. Superconductivity.

2.1 Nambu Green's functions and Gorkov equations

We derive the Gorkov's equations (GE) within the functional integral approach starting from the effective electron action for grassmanian fields ψ^{*X}, ψ^Y.

$$\mathcal{A} = \frac{1}{T} \left[\psi^{*X} \left(g^{-1}\right)^{XY} \psi^Y + \frac{1}{2} \psi^{*Y} \psi^Y V^{YX} \psi^{*X} \psi^X \right] \tag{13}$$

where X, Y denote space coordinate, sublattices (pseudospin) and spin of the electron. Finite temperature properties of the condensate are described at temperature T by the normal and the anomalous Matsubara Greens functions (GF) for spin singlet state. Two equations of the Grossmanian field can be obtained by standard method (see[35] of the text and references therein)

$$\left\langle \psi^A \psi^{*B} \right\rangle \frac{\delta}{\delta \psi^{*C}} \left\langle \frac{\delta A}{\delta \psi^{*B}} \right\rangle + \left\langle \psi^A \psi^B \right\rangle \frac{\delta}{\delta \psi^{*C}} \left\langle \frac{\delta A}{\delta \psi^B} \right\rangle = 0 \tag{14}$$

$$\left\langle \psi^A \psi^{*B} \right\rangle \frac{\delta}{\delta \psi^C} \left\langle \frac{\delta A}{\delta \psi^{*B}} \right\rangle + \left\langle \psi^A \psi^B \right\rangle \frac{\delta}{\delta \psi^C} \left\langle \frac{\delta A}{\delta \psi^B} \right\rangle = \delta^{AC} \tag{15}$$

Using the definition of the normal and anomalous Green functions in the form

$$F^{AB} = \left\langle \psi^A \psi^B \right\rangle ; G^{AB} = \left\langle \psi^A \psi^{*B} \right\rangle \tag{16}$$

one obtains two Gorkov equations in the matrix form

$$F^{AX} \left[\left(g^{-1} \right)^{CX} - v^{XC} G^{CX} + v^{CX} G^{XX} \right] + G^{AX} v^{XC} F^{XC} = 0 \tag{17}$$

$$G^{AB} - G^{AX} \Delta^{XY} g^{ZY} \Delta^{*ZU} g^{UB} = g^{AB} \tag{18}$$

Here g^{UB} is the free electron gas Green function.

Skipping second and third terms in bracket of the first equation (as proportional to electron-electron interaction term) in this expression and defining, superconducting gap

$$\Delta^{AB} = v^{AB} F^{AB} \tag{19}$$

one rewrites as a matrix product:

$$\left(g^{-1} \right)^{CX} F^{XA} = -G^{AX} v^{XC} F^{XC} = -G^{AX} \Delta^{XC} \tag{20}$$

Multiplied this equation from left by G_0 one obtains

$$F^{AB} = -G^{AX} g^{BY} \Delta^{XY} \tag{21}$$

Denoting $A = a, \sigma, A; B = b, \rho, B; X = x, \kappa, X; Y = y, \chi, Y$ where first index denotes the space variables, second is spin and third the sublattice one obtains after using the Fourier series

$$F^{\sigma\kappa AX}_{ax} = \epsilon^{\sigma\kappa} \sum_\alpha e^{i\alpha(a-x)} F^{AX}_\alpha ; \Delta^{\sigma\kappa AX}_{ax} = \sum_\alpha e^{i\alpha(a-x)} \epsilon^{\sigma\kappa} \Delta^{AX}_\alpha ; \tag{22}$$

$$G^{\sigma\kappa AX}_{ax} = \delta^{\sigma\kappa} \sum_\alpha e^{i\alpha(a-x)} G^{AX}_\alpha ; v^{\sigma\rho AB}_{ab} = \sum_\alpha e^{i\alpha(a-b)} v_\alpha \tag{23}$$

here α denotes the momentum and Matsubara frequency index.

Substituting 22 into 17 and 18 one obtain two GE in the form

$$\Delta^{AB}_\omega = -_\nu v_{\omega-\nu} G^{AX}_\nu \Delta^{XY}_\nu g^{BY}_{-\nu} \tag{24}$$

$$G^{AB}_\alpha + G^{AX}_\alpha \Delta^{XY}_\alpha g^{ZY}_{-\alpha} \Delta^{*ZU}_{-\alpha} g^{UB}_\alpha = g^{AB}_\alpha \tag{25}$$

In the sublattice matrix form

$$G^{AB}_\alpha + G^{AX}_\alpha \Delta^{XY}_\alpha g^{tYZ}_{-\alpha} \Delta^{*ZU}_{-\alpha} g^{UB}_\alpha = g^{AB}_\alpha \tag{26}$$

$$G_\alpha \left\{ I + \Delta_\alpha g^t_{-\alpha} \Delta^*_{-\alpha} g_\alpha \right\} = g_\alpha \to G_\alpha = g_\alpha \left\{ I + \Delta_\alpha g^t_{-\alpha} \Delta^*_{-\alpha} g_\alpha \right\}^{-1}$$

2.2 Gap equation

Convoluting the first GE by v_ν^{AB} one obtains in matrix form

$$\Delta_\omega = -_\nu v_{\omega-\nu} G_\nu \Delta_\nu g_{-\nu}^t \tag{27}$$

Using the solution of the second GE for the Green function G

$$G_\alpha = g_\alpha \left\{ I + \Delta_\alpha g_{-\alpha}^t \Delta_{-\alpha}^* g_\alpha \right\}^{-1} \tag{28}$$

and substituting it into the first GE one obtains

$$\Delta_\omega = -_\nu v_{\omega-\nu} g_\nu \left\{ I + \Delta_\nu g_{-\nu}^t \Delta_{-\nu}^* g_\nu \right\}^{-1} \Delta_\nu g_{-\nu}^t \tag{29}$$

where $\omega = \{\mathbf{q}n\}, \nu = \{\mathbf{p}m\}$;

In the case of conventional electron gas with

$$g_\nu^{-1} = i\nu - \varepsilon_p \tag{30}$$

one obtains

$$\Delta_\omega = -_\nu v_{\omega-\nu} \frac{\Delta_\nu}{g_\nu^{-1} g_{-\nu}^{-1} + \Delta_\nu \Delta_{-\nu}^*} = -_\nu v_{\omega-\nu} \frac{1}{(i\nu - \varepsilon_p)(-i\nu - \varepsilon_p) + \Delta_\nu \Delta_{-\nu}^*} \Delta_\nu \tag{31}$$

the usual gap equation

$$\Delta_\omega =_\nu v_{\omega-\nu} \frac{\Delta_\nu}{\nu^2 + \varepsilon_p^2 + \Delta_\nu \Delta_{-\nu}^*}. \tag{32}$$
